# Association between Cognitive Distortion, Type D Personality, Family Environment, and Depression in Chinese Adolescents

**DOI:** 10.1155/2011/143045

**Published:** 2011-07-02

**Authors:** Yong Zhang, Hengfen Li, Shaohong Zou

**Affiliations:** ^1^Center of Mental Health, Tianjin 300222, China; ^2^Mental Health Institute of the Second Xiangya Hospital, Central South University, Changsha 410011, China

## Abstract

*Purpose*. Depression prevalence and risk increase among adolescents are related to biological, psychosocial, and cultural factors. Little is known about the association between cognitive distortion, type D personality, family environment, and depression. The aim of this paper was to examine the relationships of cognitive distortion, type D personality, family environment, and depression in a sample of Chinese adolescents. *Methods*. A sample of Chinese adolescents with depression and the controls were investigated cross-sectionally with life orientation test-revised (LOT-R), type D personality Scale-14 (*DS14*), family environment scale (FES), and Zung self-depression scale (SDS); respectively, all scales were administered in Chinese. *Results*. Chinese-depressed adolescents showed more cognitive distortion, type D personality, and adverse family environment than control groups. Furthermore, lower level of Optimism, negative affectivity, and poor family cohesion may increase the risk of depression in Chinese adolescents. *Conclusions*. Our study indicates that lower level of Optimism, Negative Affectivity, and poor Family Cohesion factors were implicated to contribute to depression in Chinese adolescents. Lower level of optimism and negative affectivity may be crucial associated factors of depression among these samples. our findings pointed to the importance of broad screening and intervention of vulnerable population.

## 1. Introduction

Depression is a recurrent, common disorder during adolescence and may occur more often at this time than any other life stage [1, 2]. Cross-sectional study showed that elevated depressive symptoms were reported by 40% of girls and 30% of boys. Socioeconomic status, race, and age group were independently associated with depressive symptomatology [3]. Prospective longitudinal studies show that average levels of depressive symptoms rise substantially in middle adolescence [4]. Some of adolescents who reported depressive symptoms may recover in the later life; actually prevalence of depression in adolescents was estimated less than 10% [5, 6]. These studies are from western culture; however, studies have noted differences in the prevalence and associated factors for adolescents' depression among races, gender, and cultures. Some researches reported that occurrence of depression in adolescents might be associated with different race and cultural background [7, 8]. Auerbach et al. found that Chinese adolescents reported higher level of depressive symptom than Canadian adolescents because of different culture and beliefs [9].

Depression is a multi-factorial syndrome, so associated factors need to be evaluated to provide a complete understanding of depression starting in adolescence. There are evidences showing that some psychosocial factors may increase depression risks among adolescents [10]. Some findings suggest that cognitive distortion may be associated with depression [11, 12]. Beck proposed an etiological model of depression in which maladaptive cognitive scheme place individuals at risk, conceptualizing maladaptive cognitive style as a mediator influenced on depression [13]. Research findings showed higher prevalence of depression in adolescents due to maladaptive cognitive style [14]. Kolko et al. found that depressive symptoms reduced when cognitive bias was corrected [15]. Therefore, these findings indicated that cognitive distortion may confer vulnerability to depression among adolescents.

Denollet introduced “Distressed Personality” and developed the type D personality. It is defined as the interaction of negative affectivity (the tendency to experience the negative emotions) and social inhibition (the tendency to inhibit the expression of these emotions in social interaction) [16]. Researchers have examined type D personality associated with increased risk of depressive symptoms in cardiovascular patients [17]. Little is known, however, of the relationship between depression and type D personality, particularly in Chinese adolescents. 

Family environmental factors may also contribute to the development of depressive symptoms among adolescents. Studies reported that depressed children expressed lower levels of satisfaction and cohesion, higher levels of conflict than did controls [18]. The literature indicates that low cohesion, high conflict, and high maternal control were associated with youth depression [19]. Family conflict has been found to be associated factor for youth depression [20].

While there is considerable research focusing on the relationship of depression and other correlated factors in adolescents; for example, high overprotection from parents, low self-esteem, and negatively coping style, as well as maternal depression had been investigated as predictors of youth depression [21, 22]. The current study, to our knowledge, is the first to investigate the relationship between depression, cognitive style, type D personality, family environment, and the interaction between these factors in adolescents. In a sample of Chinese-depressed adolescents and healthy controls, the current study had the following aims: (a) to compare the difference between depressed adolescents and normal controls in cognitive style, type D personality, and family environment factors, (b) to examine the relationship between depression and cognitive style, type D personality and family environment factors, and (c) to assess the relative contribution of the different associated factors to depression in Chinese adolescents.

## 2. Methods

### 2.1. Sample

Adolescent patients with initial depression were derived from the psychological clinic of Xinxiang second hospital, Xinxiang Medical College, from May to October, 2005. Diagnoses were obtained by two specialized professors according to DSM-IV criteria for depression. Depressive symptoms were assessed using the Structured Clinical Interview for the DSM-IV(SCID-I) [23], and meanwhile, the scores of Zung self-rating depression scale equal and above 50 (SDS ≥ 50). 

Ninety-four patients were screened for study, five patients were excluded due to not finishing all scales eventually, and three patients refused to participate. The final patient sample consisted of 86 patients (45 males and 41 females), with a mean age of 16.1 years (SD = 1.7). In terms of education, the sample consisted entirely of high school students. The depressed adolescents stem from three different sorts of schools: key high school, ordinary high school, and vestibule school (In China, the construction of high school include these three sorts, whose purpose lie in cultivating different school work orientation after graduation.) We built up the pool sampling in our current investigation from the previous three different sorts of high schools, including 830 healthy high school students. According to matching principal (depressed group/control group 1 : 1.5), 120 students were drawn off in pool sampling, nine were excluded due to not finished overall scales, and three were unwilling to participate in this activity. The control sample actually consisted of 108 students (61 males and 47 females), with a mean age of 16.1 years (SD = 1.6). The scores of Zung self-rating depression scale below 50 (SDS < 50) in these participants. Written informed consent was obtained from the study participants and their parents. The Ethics Committee of Xinxiang Medical College approved the study protocol.

### 2.2. Measures

All measures were administered in Chinese. At the beginning of the session, the depressed and healthy adolescents were briefly informed of the goals of this study, the questionnaires were completed anonymously, and the following measures were obtained:

#### 2.2.1. Demographic Information

Questionnaire included information about demographic and socioeconomic factors such as age, gender, parents' education levels, parents' age, family structure (intact or single parents), and household income (reflection of socioeconomic status). We need to examine relationships between these factors and depression. See Table 1.

**Life Orientation Test-Revised (LOT-R; Scheier et al., 1994) [24]**

The LOT-R is a 10-item scale that assesses subjects' expectations about their future and their general sense of optimism. The LOT-R is comprised of 3 positively worded (e.g., I'm always optimistic about my future) and 3 negatively worded items (e.g., I hardly ever expect things to go my way). Each item is rated on a five-point Likert type scale ranging from zero (strongly disagree) to four (strongly agree). The LOT-R has been shown to be a reliable and valid measure of optimism in North American [18] and Chinese samples [25]. In the current study, the LOT-R exhibited satisfactory internal consistency, with a Cronbach alpha of  .68 [26].

**Type D Personality Scale-14 (DS-14; Denollet et al., 1996) [16]**

The DS-14 is a 14-item scale that assesses type D personality. Each item is rated according to a five-point Likert scale from zero (*false*) to four (*true*). Items on the DS-14 break into two subscales: negative affectivity (NA) and social inhibition (SI). A score of 10 is the cutoff for both NA and SI. Individuals are classified as type D if both NA and SI are greater than or equal to 10. In the current study, DS-14 exhibited psychometrically sound, with a Cronbach's alpha of  .88 and test-retest reliability of  .76 [27].

**Family Environment Scale: Chinese Version (FES-CV; Moos & Moos, 1981; Chinese version: Phillips, 1999) [28]**

The FES-CV measures the social-environmental characteristics of families based on a three-dimensional conceptualization of families. It measures three different dimensions with 10 subscales, including cohesion, expressiveness, conflict, independence, achievement orientation, intellectual-cultural orientation, active recreational orientation, moral-religious emphasis, organization, and control measures. Scores for each of these 10 subscales are derived to create an overall profile of family environment. FES-CV exhibited satisfactory psychometric qualities, the internal consistency Cronbach's *α* of ten subscales range from  .24 to  .75, test-retest reliability range from  .55 to  .92 [28].

**Zung Self-Rating Depression Scale (SDS; Zung, 1976) [29]**

The SDS is 20-item scale designed to assess the level of depression for individuals. The scale consists of ten positively worded and ten negatively worded questions that rate the four common characteristics of depression: the pervasive effect, the psychological equivalents, other disturbances, and psychomotor activities. Each item on the scale is scored on a scale of 1 (little of the time) to 4 (most of the time), Scores range from 25–100, with higher scores indicating higher levels of depressive symptoms. Chinese researchers has constructed Chinese population norm [30].

### 2.3. Statistical Analyses

The chi-square test was used to examine the differences between two groups on demographic variables, such as gender, mean age, educational levels for adolescents, and parents' mean age, educational levels, family structure, and family economic status. An analysis of multiple dependent variables was employed for multiple comparisons between groups on continuous variables by performing multiple *F/t* tests. We conducted univariate analysis to compare optimism, pessimism levels, negative affectivity, social inhibition levels and all family environment variables between depressed adolescents and healthy controls. All tests used were two tailed. Pearson's correlations were used to examine the relationships between severity of depression and cognitive style, type D, and family environment factors. Hierarchical linear regression was performed to determine whether depression was associated with the above variables. Path analysis was used to determine which variable was contributed to depression.

All analyses were performed using the Statistical Program for Social Sciences (SPSS), Version 13.0 for Windows. All statistical significance was set at *P* < .05.

## 3. Results

### 3.1. Demographic Characteristics

The demographic characteristics of study sample are shown in Table 1. No statistically significant differences were found between adolescent patients and normal controls on mean age, gender distribution, education levels, parental education levels, parental mean age, family structure, and socioeconomic status.

### 3.2. Comparison of Cognitive Style, Type D, Family Environment between the Depressed Adolescents and Healthy Controls

Our first aim was to examine whether adolescents with depression have lower optimistic cognitive style, predominant type D personality, and more adverse family environment than normal controls (refer to Table 2). By comparison, we found that depressed adolescents had lower scores of optimism (*P* < .01), but higher scores of pessimism (*P* < .01), higher scores of negative affectivity and social inhibition than normal controls (*P* < .01). We also found that they experienced more adverse family environment. They had lower scores of cohesion, expressiveness, intellectual-cultural, active-recreational, moral-religious emphasis, but higher score of conflict than normal controls (*P* < .01). However, comparison of the depressed adolescents and normal controls groups yielded no significant differences in Achievement Orientation, Independence and Control (*P* > .05) the result was presented as Table 2.

### 3.3. Correlation between Depression and Cognitive Distortion, Type D Personality, Family Environment, Socioeconomic Status

Our second aim was to explore the relationship between the severity of depression and cognitive distortion, type D personality, adverse family environment, and socioeconomic status. Pearson correlation analysis between depression severity and cognitive distortion, Type D personality, family environment and socioeconomic status was reported as follows: depression severity negatively related to Optimism (*r* = −0.77, *P* < .01), Cohesion (*r* = −0.52, *P* < .01), Intellectual-Cultural (*r* = −0.38, *P* < .01), Active-Recreational (*r* = −0.39, *P* < .01), Organization (*r* = −0.38, *P* < .01), and Moral-Religious Emphasis (*r* = −0.29, *P* < .01), but positively related to Negative Affective (*r* = 0.74, *P* < .01), Social Inhibition (*r* = 0.58, *P* < .01), and Conflict (*r* = 0.38, *P* < .01). There was no significant correlation between depression severity and pessimism, Independence, Achievement Orientation, Control as well as age, gender, education level, parental age, parental education level, family structure, and family income (*P* > .05).

### 3.4. Hierarchical Regression Analysis of Different Variables

In order to confirm whether Optimism, Type D personality, Cohesion in family environment factors may influence of depression, we performed hierarchical linear regression to analyze the effect of different variables on depression (refer to Table 3). Independent variables were Optimism, Pessimism, Negative Affective, and Social Inhibition, as well as all family environment factors. Four variables were shown to impact on depression: Optimism, Negative Affective, Cohesion, and intellectual-cultural factor, respectively. The model explained 66.8% of the variance (*F* = 5.71, *P* = .018). the result was presented as Table 3.

### 3.5. Path Analysis of Depression

**Figure 1:**
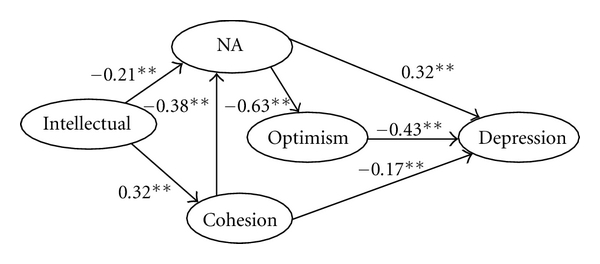
Path analysis of depression in adolescents. Note: the figure represented *B*, **represented significance level (*P* < .01).

A path analysis was conducted to determine which factor directly influenced depression, which one indirectly influenced it (refer to Figure 1). All multivariate factors, in turn, were brought into a structural equation model of depression. We confirmed major five paths that could influence occurrence of depression in adolescents:

(1) optimism → depression, (2) NA → depression, (3) NA → optimism → depression, (4) cohesion → NA → depression, (5) cohesion → NA → optimism → depression.

## 4. Discussion

To date, few studies have been conducted generally on cognitive distortion, type D personality, and family environment in adolescents with depression. The current study investigated the relationship between different associated factors and depression in a sample of Chinese adolescents. Firstly, we aimed to study the association between cognitive distortion and depression in Chinese adolescents. Consistent with previous findings [31, 32], our findings indicated that Chinese-depressed adolescents showed lower levels of optimism than normal controls and showed negative correlation between optimism and depression in Chinese adolescents. Rapaport and colleagues reported that minor depression was characterized by cognitive symptoms [33]. Furthermore, Marton and Kutcher revealed that cognitive distortion was associated with more severe symptoms of depression [14]. It is possible that cognitive distortion be vulnerability to depression regardless of severity of its symptoms [34]. Our findings confirmed that lower optimism was associated with depression and may be one of risks of depression. We found that Chinese adolescents are likely to experience school and academic achievement stress, they could show pessimism because of study pressure. Furthermore, evidences showed that this negative cognitive process possibly was more common in depressed adolescents when they may face more stress [35, 36]. Beck proposed an etiological model of depression in which maladaptive cognitive schemas may predict depression [13]. Timbremont and Braet (2004) reported that depressive symptoms of adolescents may relapse if their vulnerable cognitive process could not be changed [37]. It seems to demonstrate that cognitive vulnerability factor increases in depression.

Another finding in our study suggests that Chinese-depressed adolescents display obviously type D personality comparing to normal controls. Negative affectivity (NA) and social inhibition (SI) are two traits of type D. Our findings indicate that Chinese adolescents with type D personality trait were prone to depressive symptoms, type D was related to increased risk of depression, in addition, NA and SI showed positive correlation with depression, and NA characteristic was significantly associated with depression. The depressed adolescents with type D personality tended to perceive distress emotion and experience unhappy life events. Their social activities may be prone to self inhibition due to worry refusal in communication. Pedersen et al. proved that type D personalities were associated with close to a 3-fold increased risk of developing depressive symptoms [38]. We confirmed that type D personality factors may increase the risk of depression among these populations.

Our study revealed that poor family cohesion and conflict were associated with increased risks of depression in Chinese adolescents. Chinese researches (Liu et al., 2008) found that low cohesion (OR: 3.07, 95%CI: 1.67–5.67) and high conflict (OR: 4.94, 95%CI: 2.92–8.37) may predict suicidal ideation or attempt of Chinese adolescents [39]. Our study indicated that poor cohesion as well as family conflict may correlate with depression. Besides, we demonstrated that expressiveness, intellectual-cultural, active-recreational, organization and moral-religious emphasis variables to extent were related to depression in Chinese adolescents but did not find the correlation of achievement, independence, control variables, and depression. Reinherz et al. argued that achievement, independence, and Organization variables were the significant risk factor for depression in adolescents [40]. Alloy et al. considered expressiveness Control were all important predictors of depression [41, 42]. Possible reasons for different findings might be that most Chinese parents regard children as their own properties, and children have to obey the rules made by parents. Chinese parents may ignore communication with their children but be more likely to request that their children focus on only school courses. Therefore, these adolescents, especially male adolescents confronted to double pressures from parents and schools, for instance, entering a higher or key school was the most important goal parents and school required. On the other hand, Chinese adolescents regard their parents supervising as restriction and control, Consequently, they expressed more conflict with parents, poor expressiveness and cohesion, tired of school work, and prone to depression. This possibly contributed to different cultural background and relief between Chinese family and western family [43].

We constructed depression diathesis model to examine the contribution to depression. Path analysis indicated that optimism variable had direct impact on depression, and it had stronger contribution to depression. However, while NA and cohesion variable also had a direct impact on depression, their respective contributed values were minor. In addition, we found that NA had a stronger impact on optimism; we speculated that NA as a predisposition may indirectly influence depression. And cohesion had a impact on NA, showing an important influence of family environment on development of adolescent personality. Generally, the interaction of all variables leads to an understanding of the multifactorial nature of depression [44].

Our study has numerous limitations. Firstly, our patient samples were recruited from hospital clinic rather than community; the depressed and healthy controls were almost drawn from urban area and cannot be considered representative of the all adolescent population, we did not investigate adolescents from rural area. Some researchers found that Chinese adolescents from rural area reported more likely to depression even suicidality [45]. Our sample only investigated students in schools; some dropouts, particularly adolescents who had no opportunity to enter school from rural area, were not included of our investigation; this may lead to an underestimation of depressive symptoms. Secondly, although we assessed general depressive symptoms based on depression diagnoses (Zung SDS ≥ 50), we did not differentiate between major depressive disorder (MDD) and minor depressive disorder, and little is known about difference between MDD and minor depressive disorder with regard to cognitive style, type D, and family variables. Researchers have reported rate of MDD (25%) as higher than that of minor depressive disorder (9.9%) [46], and MDD was characterized by classical neurovegetative symptoms while minor depressive disorder was characterized by cognitive symptom [33, 47]. It is necessary to examine the impact of multifactorial risks on the two subtypes of depressive disorder. Thirdly, although our study found some associated factors of depression, their impact value was not strong, and we did not describe comorbidities presented from SCID (e.g., anxiety, etc.). Other psychiatric disorders might also be correlated with the LOT, DS, and FES scales; therefore we prudently concluded our results. Other related factors or broader presentation picture on ground of large sample should be considered carefully.

In summary, our findings indicate that Chinese-depressed adolescents display obviously low levels of optimism, type D personality, family conflict, and poor family cohesion. Of these risks, low levels of optimism and negative affectivity may significantly be associated with depression in Chinese adolescents. The findings from current study seem to suggest low levels of optimism, negative affectivity, and poor family cohesion contributed to depression among Chinese adolescents. Our study may provide some meaningful methods to evaluate the depression in adolescents, we should sufficiently pay attention on these increasing risks in course of targeted interventions and preventive efforts in this population.

## Figures and Tables

**Table 1:** Demographic characteristics of study sample.

	Depression (*N* = 86)	Normal Controls (*N* = 108)	*χ*2/*t*	*P*
*Age*	16.1 ± 1.7	16.1 ± 1.6	6.380	.380

*Sex*			.334	.663
male	45 (52.3%)	61 (56.5%)		
female	41 (47.7%)	47 (43.5%)		

*Education*			4.339	.051
middle school	38 (44.2%)	32 (29.6%)		
high school	48 (55.8%)	76 (70.4%)		

*Father education *			1.627	.435
≤middle school	26 (30.2%)	25 (23.1%)		
high school	36 (41.9%)	54 (50.0%)		
>high school	24 (27.9%)	29 (26.9%)		

*Mother education *			2.026	.369
≤middle school	35 (40.7%)	36 (33.3%)		
high school	39 (45.3%)	60 (55.6%)		
>high school	12 (14.0%)	12 (11.1%)		

*Father age*	43.7 ± 4.4	42.7 ± 3.8	33.863	.129

*Mother age *	42.9 ± 4.5	41.2 ± 3.4	32.654	.136

*Family structure*			.312	1.000
Two parents	82 (95.3%)	102 (94.4%)		
Single parent	1 (1.2%)	2 (1.9%)		
Neither-parent-living	3 (3.5%)	4 (3.7%)		

*Family income*			1.200	.630
Below average	7 (8.1%)	9 (8.3%)		
Average	77 (89.5%)	93 (88.0%)		
Above average	2 (2.4%)	6 (3.7%)		

Note: Chi-Square test adopted Fisher's value of exact probability; % represented composing percent.

**Table 2:** Comparison of LOT, DS, and FES between two groups $\left(\overset{̅}{x}\pm s\right)$.

Factor	Depression (*N* = 86)	Normal controls (*N* = 108)	*t*	*P*
Optimism	7.62 ± 3.20	12.02 ± 2.07	−11.59	.000
Pessimism	7.54 ± 1.98	6.59 ± 1.88	3.37	.001
LOT-total	14.21 ± 3.74	19.56 ± 2.76	−11.45	.000
Negative affective	14.83 ± 6.18	5.89 ± 4.55	11.60	.000
Social-Inhibition	14.90 ± 6.04	8.52 ± 5.16	7.92	.000
DS-Total	29.72 ± 11.29	14.41 ± 8.36	10.85	.000
Cohesion	5.56 ± 2.66	7.44 ± 1.81	−5.83	.000
Expressiveness	4.23 ± 1.86	5.13 ± 1.75	−2.59	.011
Conflict	4.48 ± 2.78	3.17 ± 1.97	3.84	.000
Independence	5.08 ± 1.58	4.84 ± 1.54	1.06	.291
Achievement	5.66 ± 1.71	5.81 ± 1.55	−0.65	.518
Intellectual-Cultural	3.23 ± 1.96	4.39 ± 1.96	−4.08	.000
Active-Recreational	2.98 ± 2.20	4.74 ± 2.23	−5.51	.000
Moral-Religious	4.52 ± 1.69	5.24 ± 1.39	−3.25	.001
Organization	4.86 ± 1.87	5.94 ± 1.85	−4.00	.000
Control	3.14 ± 1.80	3.36 ± 1.85	−0.84	.403

Note: statistical significance was set at *P* < .05.

**Table 3:** Hierarchical regression analysis of significant factors predicting depression.

Model	Adjusted *R* ^2^	*B*	SE	*β*	*t*	*P* value
Model 1	.589					
optimism		−2.281	.137	−.769	−16.650	.000

Model 2	.641					
optimism		−1.443	.201	−.486	−7.176	.000
NA		.535	.099	.366	5.400	.000

Model 3	.660					
optimism		−1.322	.199	−.445	−6.637	.000
NA		.468	.099	.320	4.752	.000
Cohesion		−.685	.294	−.163	−3.357	.001

Model 4	.668					
optimism		−1.265	.198	−.426	−6.385	.000
NA		.461	.097	.315	4.735	.000
Cohesion		−.587	.206	−.140	−2.857	.005
Intellectual-cultural		−.531	.222	−.107	−2.389	.018

Note**: **NA is negative affectivity.
